# Targeted therapy for mucinous ovarian carcinoma: evidence from clinical trials

**DOI:** 10.1136/ijgc-2022-003658

**Published:** 2022-09-06

**Authors:** Devindee Nugawela, Kylie L Gorringe

**Affiliations:** 1 The University of Melbourne, Melbourne, Victoria, Australia; 2 Sir Peter MacCallum Dept of Oncology, The University of Melbourne, Melbourne, Victoria, Australia; 3 Peter MacCallum Cancer Centre, Melbourne, Victoria, Australia

**Keywords:** cystadenocarcinoma, mucinous, medical oncology

## Abstract

Mucinous ovarian carcinoma is a rare subtype of epithelial ovarian cancer. Despite being a chemoresistant tumour type, surgical resection and chemotherapy are still the current standard for management. This narrative review aims to explore the current evidence for targeted therapies in mucinous ovarian carcinoma. A review of the literature was performed to identify clinical trials and case reports of targeted therapy in patients with mucinous ovarian carcinoma. The databases and registers (PubMed, MEDLINE, Embase, Europe PMC, Cochrane Central Register of Clinical Trials, clinicaltrials.gov) were searched for articles published between January 2009 to June 2021 using keywords specific for mucinous ovarian carcinoma and targeted therapy. Records were screened and assessed for eligibility based on inclusion and exclusion criteria. From 684 records, 21 studies met the criteria to be included in the review. A total of 11 different targeted therapies were identified, each demonstrating varying degrees of clinical evidence supporting further investigation in patients with mucinous ovarian carcinoma. Targeted therapies identified in this review that warrant further investigations are bevacizumab, trastuzumab, nintedanib, AZD1775, sunitinib, cediranib and pazopanib. Many of the therapeutic agents may be investigated further in combination with other targeted therapies or chemotherapy. More clinical trials focusing on targeted therapy specifically in patients with mucinous ovarian cancer are required to inform clinical use. Multinational efforts are likely to be required to successfully conduct trials in this rare tumor type.

## Introduction

Mucinous ovarian carcinoma accounts for 3–5% of all epithelial ovarian cancers.^1^ Current standard therapeutic management involves surgical resection and platinum/taxane chemotherapy, similar to that employed in high-grade serous ovarian carcinoma.^2^ Curative outcomes may be achieved with surgical resection if diagnosis is made at an early stage. However, mucinous ovarian carcinomas, especially if recurrent, with advanced spread or high grade, are chemoresistant.^3^ Median survival of stage III/IV disease is less than 15 months, compared with 41 months for high-grade serous ovarian carcinoma.^4^ Patients with mucinous ovarian carcinoma are currently disadvantaged since many of the treatment regimens they are offered do not have the same level of evidence as for other cancers.^5^ Given these poor outcomes, new therapeutic strategies are needed for this chemoresistant tumor type.

Targeted therapy involves agents that target cancer-associated molecules to specifically inhibit the growth and spread of malignant cells. In contrast, chemotherapy uses cytotoxic drugs that kill both cancer cells and normal cells, hence, patients are likely to experience more toxic adverse effects.^6^ Gene mutations that confer alterations to cellular proteins and receptors form one of the hallmarks of carcinogenesis. Molecular alterations that occur in mucinous ovarian carcinoma include *KRAS/NRAS* mutations (65.8%), *TP53* mutation (65.2%), *ERBB2* amplification (26.7%) and *BRAF* mutation (8.7%).^1^ These gene mutations and the resulting proteins can be used to distinguish between normal cells and cancer cells and can serve as specific molecular targets for targeted therapy. Targeted therapy can also impact key molecular drivers that promote the growth, survival and spread of malignant cells such as vascular endothelial growth factor (VEGF). This targeting can be direct, as in the case of imatinib for BCR-ABL1 fusions or indirect, by targeting a pathway synergy, as in poly adenosine diphosphate-ribose polymerase (PARP) inhibitors for *BRCA1/2*-mutated tumors. The highly specific nature of targeted therapy suggests that these agents are likely to use mechanisms of action that evade the chemoresistant features of mucinous ovarian carcinoma cells. Examples of targeted agents include monoclonal antibodies or small molecules that target specific tumour-associated antigens, tumour-promoting molecules or against immune checkpoint proteins. Therapeutic agents targeting specific biomarkers and key molecular drivers of mucinous ovarian carcinoma such as *ERBB2* and *KRAS* may exhibit promising results.^7^

**Figure 1:**
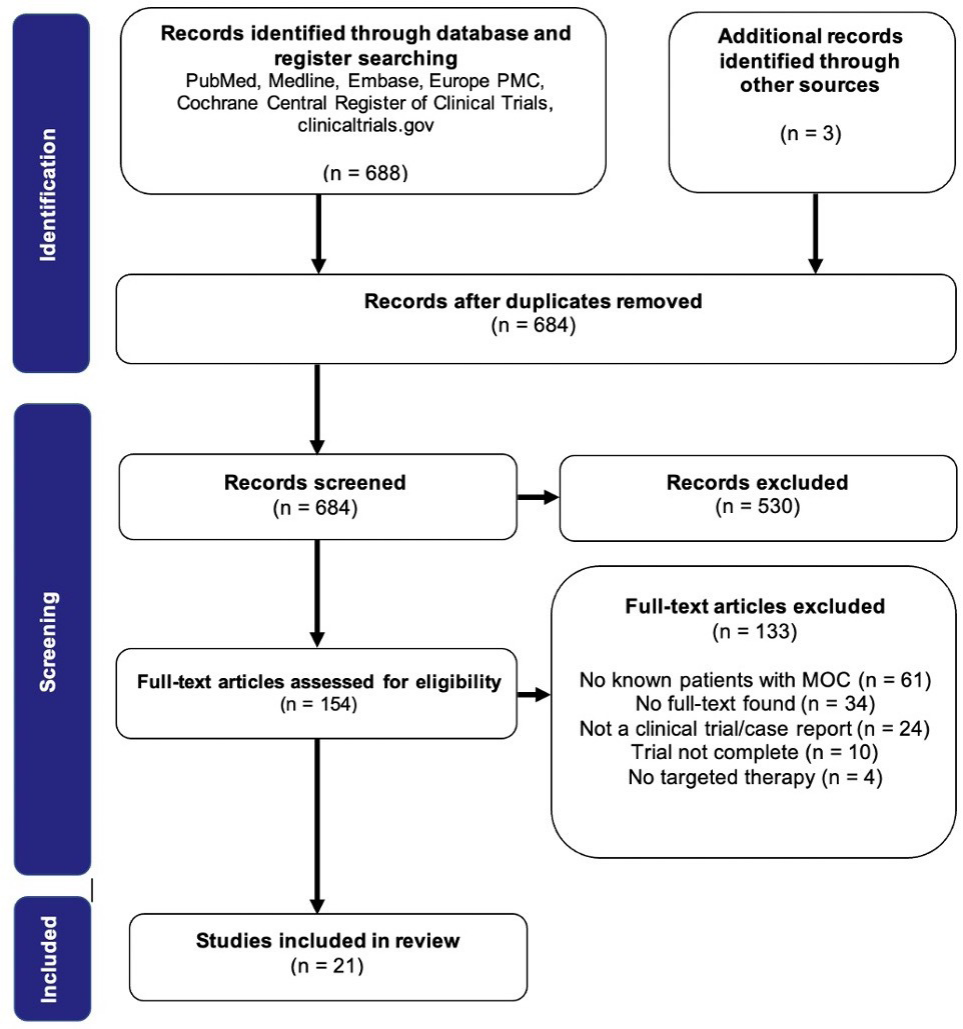
Preferred Reporting Items for Systematic Reviews and Meta-Analyses (PRISMA) diagram outlining the review process. Records were screened based on the inclusion and exclusion criteria determined for this review. Papers were only included if they were clinical trials or case reports, included patients with mucinous ovarian carcinoma and targeted therapy was at least one of the interventions. Papers were excluded if they were published before 2009, full text was unavailable or if the full text was not available in English. Studies that reported outcomes based on tumor markers, tumor burden on imaging, survival analysis (overall survival, progression-free survival), response evaluation criteria in solid tumors (RECIST) response criteria or Eastern Cooperative Oncology Group (ECOG) performance status were considered relevant.

This narrative review aims to evaluate the literature to identify and appraise the outcomes of clinical trials of targeted therapy that have included patients diagnosed with advanced (stage II-IV) or recurrent mucinous ovarian carcinoma. Currently, clinical trials of targeted therapy specifically for mucinous ovarian carcinoma have not been completed due to the rarity of the cancer. For instance, the rare tumor trial GOG-0241 evaluating four treatment regimens for mucinous ovarian carcinoma was terminated early due to poor accrual.^5^ However, patients with this disease are likely to have been included in pan-ovarian or pan-cancer clinical trials with a specific tumor biomarker determining trial entry. We performed a systematic literature and database search to identify such studies (Figure 1) and report those that included mucinous ovarian carcinoma grouped by the biological pathway targeted (Table 1). Through the evaluation of current evidence for novel therapeutic approaches, we aim for this study to contribute to offering personalized therapies to patients with mucinous ovarian carcinoma, stratified based on their tumor biomarkers.

**Table 1:** Summary of the included studies

Author(s)	Year	Type of study	Targeted therapy	Types of cancers eligible	Total recruited	No. of mucinous ovarian carcinoma	No. of mucinous ovarian carcinoma patients per study arm	Overall study outcomes
Gore et al^5^	2019	Phase III	Bevacizumab	Stage II - IV or recurrent mucinous ovarian carcinoma.	50	18*	Bevacizumab - 24 No bevacizumab −26	Trial terminated due to poor accrual.
Burger et al^8^	2011	Phase III	Bevacizumab	Stage III/IV epithelial ovarian, primary peritoneal, or fallopian-tube cancer.	1873	19	Bevacizumab initiation - 5 Bevacizumab through - 8 No bevacizumab - 6	Progression-free survival (PFS) improved by 4 months with bevacizumab/chemotherapy compared with chemotherapy alone.
Perren et al^9^	2011	Phase III	Bevacizumab	Stage IIB -IV epithelial ovarian, primary peritoneal, or fallopian-tube cancer.	1528	15	Bevacizumab – 19 No bevacizumab – 15	Improved PFS by 2 months in the chemotherapy and bevacizumab treatment group.
Aghajanian et al^10^	2012	Phase III (OCEANS)	Bevacizumab	Recurrent ovarian, fallopian tube or primary peritoneal cancer.	484	4	Bevacizumab – 3 No bevacizumab – 1	Improved PFS in chemotherapy/ bevacizumab compared with chemotherapy alone.
Coleman et al^11^	2017	Phase III	Bevacizumab	Recurrent ovarian, fallopian tube or primary peritoneal cancer.	674	4	Bevacizumab - 2 No bevacizumab - 2	Improved overall survival (OS) by 5 months in the chemotherapy and bevacizumab group.
Winer et al^12^	2010	Case report	Bevacizumab	–	–	1	–	Stable disease for 9 months before disease recurrence. Achieved stable disease again after reinitiating bevacizumab.
Tarumi et al^13^	2017	Case report	Bevacizumab	–	–	1	–	Responded with stable disease and improved ECOG performance status.
Ledermann et al^14^	2016	Phase III (ICON6)	Cediranib	Recurrent ovarian, fallopian tube or primary peritoneal cancer.	456	4	Maintenance - 1 Concurrent - 3 No cediranib - 0	Improved PFS – 11.0 months for maintenance cediranib vs 8.7 months for chemotherapy /no maintenance therapy.
Baumann et al^15^	2012	Phase II	Sunitinib	Epithelial ovarian, primary peritoneal or fallopian tube cancer.	73	3	Non-continuous sunitinib - 1 Continuous sunitinib - 2	Higher response rate for non-continuous sunitinib. No significant difference observed in PFS or OS between the two arms (continuous and non-continuous therapy).
du Bois et al^16^ Ray-Coquard et al^17^	2016, 2019	Phase III (AGO-OVAR12)	Nintedanib	Stage IIB–IV epithelial ovarian, fallopian tube, or primary peritoneal cancer.	1366	37	Nintedanib - 25 No Nintedanib - 12	Significant improvement in PFS. No improvement in median OS – placebo better than nintedanib in patients with mucinous ovarian carcinoma.
Vergote et al^18^	2019	Phase III (AGO-OVAR16)	Pazopanib	Stage II–IV epithelial ovarian, fallopian tube, or primary peritoneal cancer.	940	40	Pazopanib - 24 No pazopanib −16	Prolonged PFS – 17.9 months for pazopanib vs 12.3 months for placebo. No improvement in median OS.
Richardson et al^19^	2018	Phase II	Pazopanib	Recurrent epithelial ovarian, fallopian tube, or primary peritoneal cancer.	106	1	Pazopanib - 1 No pazopanib - 0	No improvement in PFS or OS.
McAlpine et al^20^	2009	Case report	Trastuzumab	–	–	2	–	Both achieved stable disease. Disease recurred when therapy ceased. One patient improved with re-initiation of trastuzumab.
Jain et al^21^	2012	Case report	Trastuzumab	–	–	1	–	Downtrend in the serum marker CEA and improved ECOG performance status.
Benjamin et al^22^	2014	Case report	Trastuzumab	–	–	1	–	Progressive disease.
Vergote et al^25^	2014	Phase III	Erlotinib	Stage II - IV epithelial ovarian, primary peritoneal, or fallopian tube cancer.	835	14	Erlotinib – 6 No erlotinib – 8	No improvement in PFS or OS.
Leijen et al^27^	2016	Phase II	AZD1775	Recurrent epithelial ovarian cancer (with *TP53* mutation).	23	2	No randomized arms	One mucinous ovarian carcinoma patient achieved partial response and the other patient had progressive disease.
Sabbatini et al^28^	2013	Phase III	Abagovomab	Stage III - IV epithelial ovarian, fallopian tube, or primary peritoneal cancer.	888	9	Abagovomab – 6 No abagovomab – 3	No improvement in PFS or OS observed.
Schilder et al^31^	2012	Phase II	Dasatinib	Epithelial ovarian, primary peritoneal cancer.	34	1	No randomized arms	No improvement in PFS.
Meier et al^33^	2012	Phase II	Lonafarnib	Stage IIB - IV epithelial ovarian, fallopian tube, or primary peritoneal cancer.	105	5	Lonafarnib – 1 No lonafarnib – 4	No improvement in PFS or OS.

*50 patients recruited initially, only 18 confirmed to have mucinous ovarian carcinoma.

### Targeted Therapy Agents

#### VEGF Inhibitors

The principle mechanism of action of monoclonal VEGF antibodies is via the inhibition of tumor angiogenesis. In four phase III ovarian cancer trials, including a total of 42 patients with mucinous ovarian carcinoma, the addition of bevacizumab to chemotherapy significantly improved overall progression-free survival by 2–4 months and median overall survival by 5 months.^8–11^ Only a subset of patients in these trials had a diagnosis of mucinous ovarian carcinoma and histological subtype-based analyses of outcomes were not reported. This limits the inferences that can be drawn regarding progression-free survival and overall survival for patients with this disease treated with bevacizumab in these trials. The rare tumor trial GOG-0241 attempted to evaluate several treatment regimens, including bevacizumab, specifically in patients with advanced mucinous ovarian carcinoma.^5^ There was no benefit to overall survival when comparing the arms with and without bevacizumab for all 50 patients (hazard ratio [HR] 1.04) as well as for the 18 patients with confirmed mucinous ovarian carcinoma after central pathology review (HR 1.08). No significant conclusions can be drawn from this study due to the small sample size. Two case reports of patients with heavily treated recurrent stage IIIa mucinous ovarian carcinoma commenced on bevacizumab resulting in resolution of symptoms and halted disease progression for 12 and 30 months respectively based on CA-125 levels, corresponding to stable disease based on response evaluation criteria in solid tumors (RECIST) criteria.^12 13^ The positive outcomes outlined on these case reports and the significant improvements in overall progression-free survival and overall survival observed in the bevacizumab trials above support further investigation of bevacizumab in a larger cohort of patients with mucinous ovarian carcinoma.

An alternative mechanism to inhibit VEGF receptors is by the small molecule inhibitor cediranib, which similarly functions by suppressing tumor angiogenesis. The phase III trial ICON6 included 4 patients with mucinous ovarian carcinoma and showed a prolongation in progression-free survival – 11 months in the group that received chemotherapy followed by maintenance cediranib, compared with 8.7 months for chemotherapy only.^14^ Overall survival data from this trial available at the time of publication, although immature, demonstrated no significant difference in overall survival in the randomized arms of the trial. Mucinous ovarian carcinoma-specific data was not reported.

#### Tyrosine Kinase Receptor Inhibitors

Platelet derived growth factor (PDGF), similar to VEGF, plays a major role in tumor angiogenesis and tumor cell proliferation. Sunitinib targets multiple receptor tyrosine kinases including the receptors for PDGF and VEGF, thereby inhibiting tumor vascularization and promoting tumor cell apoptosis. In a phase II trial comparing continuous and non-continuous monotherapy with sunitinib in platinum resistant ovarian cancer, the non-continuous regimen demonstrated the best reported response according to RECIST criteria. The response rate (complete and partial) for the non-continuous treatment arm was 15.7%, compared with 5.4% for the continuous treatment arm.^15^ This trial included 3 patients with mucinous ovarian carcinoma. Histological subtype-based analysis of responses was not reported which limits the inferences that can be drawn regarding the benefit of sunitinib in mucinous ovarian carcinoma. The results of this study support further investigation of non-continuous sunitinib monotherapy in patients with mucinous ovarian carcinoma.

In a phase III trial including 37 patients with mucinous ovarian carcinoma, treatment with nintedanib in combination with carboplatin and paclitaxel across all included subtypes resulted in a progression-free survival of 17.6 months with nintedanib and 16.6 months with placebo.^16 17^ However, this efficacy was not translated to overall survival. On subgroup analysis, the HR for the mucinous/clear cell histological classification showed that overall survival was more favorable in the placebo arm over the nintedanib arm with an HR 1.28 (0.63–2.61).^17^ The overall survival results of this trial do not strongly support a role for nintedanib in mucinous ovarian carcinoma.

A third inhibitor, pazopanib, is effective in treating renal cell carcinoma. The phase III trial AGO-OVAR16 comparing pazopanib in combination with chemotherapy vs chemotherapy alone demonstrated an improvement in progression-free survival. However, this did not translate to an improvement in median overall survival.^18^ In a phase II trial including 1 patient with mucinous ovarian carcinoma, adding pazopanib to paclitaxel revealed no benefit to progression-free survival or overall survival, compared with paclitaxel alone.^19^ Based on these two trials, there is limited evidence to support future trials of pazopanib in mucinous ovarian carcinoma.

#### Anti-Her2 Therapy

Human receptor epidermal growth factor 2 (*HER2*, also known as *ERBB2*) gene amplification is shown to drive tumor development and progression in 18–26% of patients with mucinous ovarian carcinoma.^20^ A case report described a patient with metastatic mucinous ovarian carcinoma treated previously with surgical resection, chemotherapy, bevacizumab and cetuximab with disease progression. The patient responded to trastuzumab monotherapy and combination therapy with Iapatinib with stable disease for approximately 9 months, evident with a downtrend in the serum marker CEA and improvement in Eastern Cooperative Oncology Group (ECOG) performance status.^21^ Furthermore, in a second case report of a patient with mucinous ovarian carcinoma pre-treated with chemotherapy and bevacizumab, the patient was switched to chemotherapy and trastuzumab after detecting a strong *ERBB2* gene amplification. After ceasing treatment on experiencing severe adverse effects including peripheral neuropathy, radiographic progression of disease with extensive metastasis was observed 12 months after the last treatment.^22^ Another case report discussed two patients with recurrent mucinous ovarian carcinoma. The first patient was treated with chemotherapy and trastuzumab followed by trastuzumab monotherapy and achieved stable disease. However, disease recurred when trastuzumab was stopped and improved with recommencement. The second patient was initially treated with chemotherapy only, which resulted in disease progression. After commencing trastuzumab monotherapy the patient responded with normal tumor markers and no clinical evidence of disease. However, central nervous system recurrence occurred 1 month after stopping trastuzumab.^20^ All patients in the case reports above demonstrated a positive response to treatment with trastuzumab. However, it is challenging to infer progression-free survival and overall survival outcomes through case reports alone, since patients with no response are seldom reported. These findings warrant further investigation of trastuzumab in mucinous ovarian carcinoma through clinical trials or an unbiased approach such as a registry analysis.

#### Epidermal Growth Factor Receptor Inhibitors

Epidermal growth factor receptor (EGFR) inhibition by erlotinib blocks tumor cell growth and initiates apoptosis in EGFR-overexpressing tumor cells.^23^ Approximately 55% to 98% of advanced epithelial ovarian carcinomas have been found to overexpress EGFR.^24^ A phase III trial including 14 patients with mucinous ovarian carcinoma exhibited no overall or histological subtype-based improvement in median progression-free survival or overall survival.^25^ Tumor EGFR status was not a criterion for selection of patients in this trial. Immunohistochemistry and mutation analyses for EGFR were conducted for a proportion of patients. This analysis revealed that the presence of *EGFR* mutations or overexpression did not predict erlotinib efficacy. Furthermore, in this study it was observed that patients with positive EGFR had significantly worse progression-free survival and overall survival when compared with patients with negative EGFR. This study revealed that EGFR may have a role as a prognostic marker in ovarian cancer which was not evident in previous studies.^26^ Based on the outcomes of this study, further investigation of erlotinib in the treatment of mucinous ovarian carcinoma is not warranted.

#### WEE1 Inhibitors

AZD1775 is an inhibitor of WEE1, which is a tyrosine kinase that regulates cell cycle progression. Inhibiting WEE1 increases the apoptotic response to DNA damage in p53 deficient tumor cells, thereby enhancing the anti-tumor activity of chemotherapy in platinum-resistant tumors. A phase II study of chemotherapy and AZD1775 in *TP53* mutated ovarian tumors demonstrated an objective response rate of about 43%.^27^ The trial included two patients with mucinous ovarian carcinoma, one demonstrated a partial response while the other patient had progressive disease. This study’s patient outcomes were presented with reference to individual histological subtypes and mutations. This allowed us to identify the responses of the two patients with mucinous ovarian carcinoma. The encouraging results of this agent for platinum-resistant tumors with p53 deficiency warrant further phase II and III clinical trials.

#### Monoclonal Antibodies against Tumor Markers

CA-125 is expressed by a significant proportion of epithelial ovarian cancers and elevated levels are associated with platinum resistance and increased tumor invasiveness. Abagovomab is a monoclonal antibody that mimics CA-125 and has been shown to induce tumor specific immune responses in preclinical and phase I/II studies. In a phase III trial comparing abagovomab against a placebo as maintenance therapy, 9 patients with mucinous ovarian carcinoma were included. Despite having strongly supporting phase II data, this trial observed no benefit across all patients in recurrence-free survival or overall survival.^28^ However, as the authors discuss, targeting multiple tumor antigens simultaneously has shown promising effects in other solid tumor trials. This may be a reasonable avenue for future trials targeting tumor antigens.

#### Targeting SRC Pathway

SRC family kinases and receptor tyrosine kinases are involved in signaling that promotes cell survival, growth, metastasis and angiogenesis.^29 30^ Preclinical studies have shown that SRC family kinases are overexpressed in mucinous ovarian carcinoma tumor cells and are thought to contribute to chemotherapy resistance.^30^ The anti-tumor effects of dasatinib through the inhibition of SRC family kinases and several receptor tyrosine kinases such as PDGF receptors results in inhibition of cell proliferation and promotion of cell death. Mouse in vivo studies revealed that combination therapy of dasatinib with oxaliplatin significantly reduced mucinous ovarian tumor weight.^30^ A phase II trial including one patient with mucinous ovarian carcinoma demonstrated no significant overall improvement in progression-free survival.^31^ Histological subtype-based analysis was not provided, therefore, specific outcomes of the patient with mucinous ovarian carcinoma cannot be deduced. The outcomes of this study provide inadequate evidence to direct further investigation of dasatinib in the mucinous histotype. However, despite the outcomes of this study on dasatinib monotherapy, it does not preclude further evaluation of combination therapy of dasatinib and chemotherapy in mucinous ovarian carcinoma given the pre-clinical evidence.

#### Farnesyltransferase Inhibitors

Farnesyltransferase is an enzyme involved in the functioning of Ras family proteins, which are critical oncogenes involved in cell proliferation. If a Ras protein is inappropriately activated, as in the case of *KRAS* mutation in mucinous ovarian carcinoma, it can result in proteins that promote uncontrolled cell growth and proliferation. The anti-tumor activity of lonafarnib, a farnesyltransferase inhibitor, is attributed to the inhibition of post-atranslational modifications that are required for Ras to function.^32^ A phase II trial including 5 patients with mucinous ovarian carcinoma demonstrated no benefit in adding lonafarnib to carboplatin and paclitaxel therapy, with no improvements seen in progression-free survival and overall survival compared with the chemotherapy only arm.^33^ Conclusions regarding the efficacy of lonafarnib in mucinous ovarian carcinoma cannot be drawn due to the absence of a histological subtype-based analysis of outcomes. However, based on the overall results of this trial, further investigation of lonafarnib in ovarian cancer is not supported.

### Limitations

The main limitations of the clinical trials included in this review are primarily a result of the rarity of mucinous ovarian carcinoma. There have been no completed clinical trials of targeted therapy to date that are specific to patients with this histotype, and only a single trial (GOG-0241) of this nature has been initiated to date before being terminated. Therefore, this review identified studies that have included patients with mucinous ovarian carcinoma. However, the sample size of patients with this disease within the discussed pan-ovarian clinical trials have been relatively low, with the maximum number of mucinous histotype patients in a single trial being 40, in the AGO-OVAR16 trial of pazopanib. The majority of the articles did not provide a histological subtype-based analysis of responses to therapy, limiting the inferences that can be drawn specifically regarding the efficacy of a therapeutic agent in patients with mucinous ovarian carcinoma. Second, in the future targeted therapies are likely to have the potential to be offered to patients with mucinous ovarian carcinoma based on tumor biomarker stratification. Except for trials and case reports in trastuzumab, AZD1775 and erlotinib, many of the trials discussed in this review did not have specific tumor biomarkers determining entry of patients into trials.

The main limitations of the review methodology includes the inability to search the full-text or supplementary files for keywords in the databases and registers searched for this review to increase the specificity of the search. This made it quite laborious to look through the baseline characteristics table of trials when screening records. This limitation also makes it likely that trials were potentially missed by the search process utilized in this review – trials that included patients with mucinous ovarian carcinoma but only mentioned this elsewhere in the full-text or in the supplementary materials. Unfortunately, performing such a search using a full-text searching tool such as Google Scholar results in tens of thousands of results, which is unfeasible to screen. Utilizing natural language processing systems may be an avenue for improving search efficacy and specificity in future reviews.^34^ An extension of this review could involve gathering histological subtype-based data and outcomes for the subset of patients with mucinous ovarian carcinoma in trials, by contacting the conducting team of individual trials. This step could allow inferences to be made regarding the efficacy of the targeted agents discussed.

## Conclusion

The aim of this review was to identify and evaluate the current evidence for targeted therapy in patients with mucinous ovarian carcinoma to inform directions for further investigation of therapeutic agents, thereby contributing to the development of personalized treatment and perhaps potential integration into standard treatment for this disease (Table 2). Despite the limitations, it is evident that the targeted therapies outlined in this review have varying degrees of evidence for further investigation in patients with mucinous ovarian carcinoma. The molecular targets that have been explored in the above trials are VEGF, HER2, WEE1 tyrosine kinase, multiple tyrosine kinase receptors, CA-125, PDGFR, FGFR, EGFR, farnesyltransferase and SRC family kinases. Based on the clinical trials and case reports evaluated, further investigation is warranted for bevacizumab, trastuzumab, AZD1775 and cediranib. Many of the agents, even those that showed no efficacy as monotherapies, may be investigated further in combination with other targeted therapies or chemotherapeutic agents. Due to the small sample sizes within the mucinous ovarian carcinoma subsets, observed differences in progression-free survival and overall survival may not meet the threshold for significance. Therefore, it is likely that outcomes based on RECIST criteria, ECOG performance status and tumor marker trends will be more useful in this context.

**Table 2:** Indications for further investigation of targeted agents based on the outcomes of this review

Indication for further investigation	Targeted agent
Further investigation warranted	Bevacizumab Cediranib Trastuzumab AZD1775
Further investigation may be warranted	Pazopanib Sunitinib
No evidence to support further investigation	Abagovomab Nintedanib Lonafarnib Erlotinib Dasatinib

Suggestions for future trials investigating the agents that show promising patient outcomes include aims to optimize timing and duration of treatment and identify potential patient/tumor factors that may predict treatment efficacy. It is likely that multinational efforts will be required to successfully conduct these rare tumor trials in mucinous ovarian carcinoma. However, given the phase III GOG-0241 trial that was terminated due to poor accrual, such trials may never be undertaken. In which case, evaluating outcomes of pan-ovarian or pan-cancer trials in reviews like this may help clinicians make informed choices regarding off-label treatment in mucinous ovarian carcinoma. In the future, prospective registry trials, adaptive trials and basket trials such as Bouquet-ENGOT-gyn2, as well as non-trial options such as multi-institutional series or regional/national databases, could play a role in gathering therapeutic evidence for such rare tumors. Analysis of existing data is challenging given variable reporting mechanisms, missing data and uncontrolled confounders, but could provide stimulus to pursue a promising therapy in a more controlled fashion.

## References

[1] Gorringe KL , Cheasley D , Wakefield MJ , et al . Therapeutic options for mucinous ovarian carcinoma. Gynecol Oncol 2020;156:552–60. 10.1016/j.ygyno.2019.12.015 31902686 PMC7056511

[2] Xu W , Rush J , Rickett K , et al . Mucinous ovarian cancer: a therapeutic review. Crit Rev Oncol Hematol 2016;102:26–36. 10.1016/j.critrevonc.2016.03.015 27083591

[3] Shimada M , Kigawa J , Ohishi Y , et al . Clinicopathological characteristics of mucinous adenocarcinoma of the ovary. Gynecol Oncol 2009;113:331–4. 10.1016/j.ygyno.2009.02.010 19275957

[4] Mackay HJ , Brady MF , Oza AM , et al . Prognostic relevance of uncommon ovarian histology in women with stage III/IV epithelial ovarian cancer. Int J Gynecol Cancer 2010;20:945–52. 10.1111/IGC.0b013e3181dd0110 20683400

[5] Gore M , Hackshaw A , Brady WE , et al . An international, phase III randomized trial in patients with mucinous epithelial ovarian cancer (mEOC/GOG 0241) with long-term follow-up: and experience of conducting a clinical trial in a rare gynecological tumor. Gynecol Oncol 2019;153:541–8. 10.1016/j.ygyno.2019.03.256 31005287 PMC6559214

[6] Lee YT , Tan YJ , Oon CE . Molecular targeted therapy: treating cancer with specificity. Eur J Pharmacol 2018;834:188–96. 10.1016/j.ejphar.2018.07.034 30031797

[7] Babaier A , Ghatage P . Mucinous cancer of the ovary: overview and current status. Diagnostics 2020;10. 10.3390/diagnostics10010052 PMC7168201 31963927

[8] Burger RA , Brady MF , Bookman MA , et al . Incorporation of bevacizumab in the primary treatment of ovarian cancer. N Engl J Med 2011;365:2473–83. 10.1056/NEJMoa1104390 22204724

[9] Perren TJ , Swart AM , Pfisterer J , et al . A phase 3 trial of bevacizumab in ovarian cancer. N Engl J Med 2011;365:2484–96. 10.1056/NEJMoa1103799 22204725

[10] Aghajanian C , Blank SV , Goff BA , et al . OCEANS: a randomized, double-blind, placebo-controlled phase III trial of chemotherapy with or without bevacizumab in patients with platinum-sensitive recurrent epithelial ovarian, primary peritoneal, or fallopian tube cancer. J Clin Oncol 2012;30:2039–45. 10.1200/JCO.2012.42.0505 22529265 PMC3646321

[11] Coleman RL , Brady MF , Herzog TJ , et al . Bevacizumab and paclitaxel-carboplatin chemotherapy and secondary cytoreduction in recurrent, platinum-sensitive ovarian cancer (NRG Oncology/Gynecologic Oncology Group study GOG-0213): a multicentre, open-label, randomised, phase 3 trial. Lancet Oncol 2017;18:779–91. 10.1016/S1470-2045(17)30279-6 28438473 PMC5715461

[12] Winer I , Buckanovich RJ . A case of progressive mucinous ovarian cancer of low malignant potential responsive to biologic therapy with bevacizumab. Gynecol Oncol 2010;116:578–9. 10.1016/j.ygyno.2009.10.068 19897232

[13] Tarumi Y , Mori T , Matsushima H , et al . Long-Term survival with bevacizumab in heavily pretreated and platinum-resistant mucinous ovarian cancer: a case report. J Obstet Gynaecol Res 2018;44:347–51. 10.1111/jog.13496 29121427

[14] Ledermann JA , Embleton AC , Raja F , et al . Cediranib in patients with relapsed platinum-sensitive ovarian cancer (ICON6): a randomised, double-blind, placebo-controlled phase 3 trial. Lancet 2016;387:1066–74. 10.1016/S0140-6736(15)01167-8 27025186

[15] Baumann KH , du Bois A , Meier W , et al . A phase II trial (AGO 2.11) in platinum-resistant ovarian cancer: a randomized multicenter trial with sunitinib (SU11248) to evaluate dosage, schedule, tolerability, toxicity and effectiveness of a multitargeted receptor tyrosine kinase inhibitor monotherapy. Ann Oncol 2012;23:2265–71. 10.1093/annonc/mds003 22377563

[16] du Bois A , Kristensen G , Ray-Coquard I , et al . Standard first-line chemotherapy with or without nintedanib for advanced ovarian cancer (AGO-OVAR 12): a randomised, double-blind, placebo-controlled phase 3 trial. Lancet Oncol 2016;17:78–89. 10.1016/S1470-2045(15)00366-6 26590673

[17] Ray-Coquard I , Cibula D , Mirza MR , et al . Final results from GCIG/ENGOT/AGO-OVAR 12, a randomised placebo-controlled phase III trial of nintedanib combined with chemotherapy for newly diagnosed advanced ovarian cancer. Int J Cancer 2020;146:439–48. 10.1002/ijc.32606 31381147

[18] Vergote I , du Bois A , Floquet A , et al . Overall survival results of AGO-OVAR16: a phase 3 study of maintenance pazopanib versus placebo in women who have not progressed after first-line chemotherapy for advanced ovarian cancer. Gynecol Oncol 2019;155:186–91. 10.1016/j.ygyno.2019.08.024 31519320

[19] Richardson DL , Sill MW , Coleman RL , et al . Paclitaxel with and without pazopanib for persistent or recurrent ovarian cancer: a randomized clinical trial. JAMA Oncol 2018;4. 10.1001/jamaoncol.2017.4218 PMC5838582 29242937

[20] McAlpine JN , Wiegand KC , Vang R , et al . Her2 overexpression and amplification is present in a subset of ovarian mucinous carcinomas and can be targeted with trastuzumab therapy. BMC Cancer 2009;9:433. 10.1186/1471-2407-9-433 20003286 PMC2803495

[21] Jain A , Ryan PD , Seiden MV . Metastatic mucinous ovarian cancer and treatment decisions based on histology and molecular markers rather than the primary location. J Natl Compr Canc Netw 2012;10:1076–80. 10.6004/jnccn.2012.0113 22956806

[22] Benjamin R , Zhai J , Morgan R , et al . Trismus and diffuse polymyalgia: an unusual presentation of recurrent metastatic ovarian cancer. BMJ Case Rep 2014;2014:bcr2013203361-bcr2013203361. 10.1136/bcr-2013-203361 PMC4025373 24835804

[23] Abdelgalil A , Al-Kahtani H , Erlotinib A-JF . Profiles of drug substances, excipients and related methodology, 2020: 93–117. 10.1016/bs.podrm.2019.10.004 32164971

[24] Ilekis JV , Connor JP , Prins GS , et al . Expression of epidermal growth factor and androgen receptors in ovarian cancer. Gynecol Oncol 1997;66:250–4. 10.1006/gyno.1997.4764 9264571

[25] Vergote IB , Jimeno A , Joly F , et al . Randomized phase III study of erlotinib versus observation in patients with no evidence of disease progression after first-line platin-based chemotherapy for ovarian carcinoma: a European organisation for research and treatment of Cancer-Gynaecological cancer group, and gynecologic cancer intergroup study. J Clin Oncol 2014;32:320–6. 10.1200/JCO.2013.50.5669 24366937

[26] Nicholson RI , Gee JMW , Harper ME . EGFR and cancer prognosis. Eur J Cancer 2001;37:9–15. 10.1016/S0959-8049(01)00231-3 11597399

[27] Leijen S , van Geel RMJM , Sonke GS , et al . Phase II study of Wee1 inhibitor AZD1775 plus carboplatin in patients with TP53-Mutated ovarian cancer refractory or resistant to first-line therapy within 3 months.. J Clin Oncol 2016;34:4354–61. 10.1200/JCO.2016.67.5942 27998224

[28] Sabbatini P , Harter P , Scambia G , et al . Abagovomab as maintenance therapy in patients with epithelial ovarian cancer: a phase III trial of the AGO OVAR, COGI, GINECO, and GEICO--the MIMOSA study. J Clin Oncol 2013;31:1554–61. 10.1200/JCO.2012.46.4057 23478059 PMC5795662

[29] Montero JC , Seoane S , Ocaña A , et al . Inhibition of Src family kinases and receptor tyrosine kinases by dasatinib: possible combinations in solid tumors. Clin Cancer Res 2011;17:5546–52. 10.1158/1078-0432.CCR-10-2616 21670084

[30] Matsuo K , Nishimura M , Bottsford-Miller JN , et al . Targeting Src in mucinous ovarian carcinoma. Clin Cancer Res 2011;17:5367–78. 10.1158/1078-0432.CCR-10-3176 21737505 PMC4028171

[31] Schilder RJ , Brady WE , Lankes HA , et al . Phase II evaluation of dasatinib in the treatment of recurrent or persistent epithelial ovarian or primary peritoneal carcinoma: a gynecologic Oncology Group study. Gynecol Oncol 2012;127:70–4. 10.1016/j.ygyno.2012.06.009 22710075 PMC3748717

[32] Taylor SA , Marrinan CH , Liu G , et al . Combining the farnesyltransferase inhibitor lonafarnib with paclitaxel results in enhanced growth inhibitory effects on human ovarian cancer models in vitro and in vivo. Gynecol Oncol 2008;109:97–106. 10.1016/j.ygyno.2007.12.013 18237771

[33] Meier W , du Bois A , Rau J , et al . Randomized phase II trial of carboplatin and paclitaxel with or without lonafarnib in first-line treatment of epithelial ovarian cancer stage IIB-IV. Gynecol Oncol 2012;126:236–40. 10.1016/j.ygyno.2012.04.050 22564713

[34] Kreimeyer K , Foster M , Pandey A , et al . Natural language processing systems for capturing and standardizing unstructured clinical information: a systematic review. J Biomed Inform 2017;73:14–29. 10.1016/j.jbi.2017.07.012 28729030 PMC6864736

